# Intelligence

**Published:** 1808-06

**Authors:** 


### INTELLIGENCE.

Professor Stromyer, of Gottingen, has published in thirty-two whole sheet tables, a systematic arrangement of the different substances which are the particular objects of chemical science, with a copious collation of synonisms in German, Latin, French, and English. The only innovation he has allowed himself, is the classing of oil, sugar, starch, gluten, and several other vegetable and animal matters, as oxides with compound radicals, consisting either of carbon and hydrogen, or carbon, hydrogen, and nitrogen. Among these he makes wax differ from fixed oil only, in being more oxided, and adipocere from fat in the same manner.

The same gentleman has likewise communicated part of the results of his chemical investigation of the union of hydrogen with metals. On the present occasion he confined himself to that of arsenic. This, he observes, succeeds best by digesting an alloy of fifteen parts of tin and one of arsenic with concentrated muriatic acid, in a retort connected with the pneumatic apparatus. He was led to this by the observation of Proust, that muriatic acid completely frees tin from arsenic; and on this occasion he convinced himself by experiments, that the fœtid hydrogen gas evolved, when the tin of the shops is dissolved in muriatic acid, is not a compound of tin and hydrogen, as Fourcroy conjectures in his Chemical System, but of arsenic and hydrogen. When arsenicated hydrogen is formed in the manner directed above, a very pure oximuriate of tin is obtained. Professor Stromeyer concludes with a remarkable experiment, showing the effect of oil of turpentine on arsenicated hydrogen gas; all the phenomena of which, however, do not appear easily explicable. Ten cubic inches of gas being confined over this essential oil, all the arsenic was separated in the course of ten hours, so as to leave the hydrogen gas pure. No perceptible deposition of metal or oxide took place, but the oil appeared viscous and milky, and after some time, small six-sided crystals, terminating in pyramids, were found adhering to the sides of the vessel. These crystals being set on fire, burned like oil of turpentine, emitting, at the same time, a very distinguishable smell of arsenious acid. A similar appearance took place on transmitting arsenicated hydrogen gas through oil of turpentine.

The Royal College of Surgeons, on the 9th instant, adjudged the Jacksonian prize for 1807, to John Hyslop, Esq. of Fenchurch Street, for the best Dissertation on "Diseases of the Eye, and its appendages, and the treatment of them." The same gentleman obtained the prize from the College in 1805, for the best Treatise on "Injuries of the Head from External Violence."

Mr. J. I. Hawkins, of Titchfield-street, has established a museum for the reception and exhibition of useful mechanical inventions and improvements. Although his own inventions constitute the leading feature of the Exhibition, yet it is not exclusively confined to these, for the inventions and improvements of others are, and will be introduced. In the list which he gives of those that are now exhibiting, or in preparation, may be noticed the following curious contrivances: A cock, by which a servant can draw no more liquor than is ordered. A machine to be towed across a river, which will at the same moment draw on paper, to any reduced scale, the exact shape of the bottom; shewing at one view the depth of water in every part, together with the width of the river. A violin to fold up for the pocket. Artificial ears to assist the deaf, which can be worn out of sight, without inconvenience.

Mr. James Phenix, of Liverpool, has invented a fire alarm. By means of the expansion of heated air, water is forced out of a glass vessel upon a piece of loaf sugar, about half an inch square, the melting of which allows a spring to escape. This spring communicates with an alarm, or rings the bell in the lobby; but one of these alarms would be requisite for every room in a house.

Dr. Satterley and Dr. Young, propose to give two Courses of Medical Lectures next winter, at the Middlesex Hospital. Dr. Satterley's will be Clinical Lectures, and any of the pupils of the hospital attending them, will have the privilege of seeing the patient whose cases are discussed. He will be assisted in the department of morbid anatomy by Mr. Cartwright. Dr. Young's Course will be on the Elements of the Medical Sciences in general, and on the Practice of Physic in particular. It has been erroneously stated, in several periodical publications, that Dr. Young had a large medical work nearly ready for the press: the mistake arose from his having been for some time engaged in the preparation of these Lectures.

Dr.

Dr. Girdlestone, of North Yarmouth, in a letter dated May 6, 1808, requests the favour of the Editors to "insert this note, which is merely to state, whoever may have been the writer, in your Journal of May, of the letter which is dated Yarmouth, and signed W. G. M. D. that it is not the production of any one of the medical men who reside in THIS Yarmouth."

Mr. Carpue will commence his Summer Course of Lectures on Anatomy and Surgery, early in June. Particulars may be known by applying to Mr. Carpue, No. 50, Dean Street, Soho.

Mr. Carmichael proposes to publish, in the course of the ensuing Summer, the second edition of his Essay upon the Effects of the Salts of Iron upon Cancer, with many additional cases, and several interesting practical observations upon that disease.—Mr. Carmichael has received some communications from practitioners, concerning their experience of those preparations, for which he begs to return his warmest thanks, and he, at the same time, takes this opportunity of earnestly requesting such of the profession as have deemed the remedy he recommended worthy of a trial, to inform him of their experience of its effects before the end of June next, in order that he may insert their observations in his Essay, and that thus the merits of the remedy may be justly appreciated.

Mr. Nicholson's new Chemical Dictionary will make its appearance about Midsummer, in one large volume, octavo.

